# Herpesvirus-Entry Mediator Inhibits the NF-*κ*B Pathway Activated by IL-17 and Fosters the Osteogenic Differentiation of Allogeneic Mesenchymal Stem Cells

**DOI:** 10.1155/2024/8146991

**Published:** 2024-08-30

**Authors:** Zhigang Rong, Yuhang Xi, Chengmin Zhang, Wei Dai, Hao Xue, Fei Luo, Jianzhong Xu, Fei Dai

**Affiliations:** Department of Orthopaedics Southwest Hospital Third Military Medical University (Army Medical University), Chongqing 400038, China

## Abstract

The challenge in developing tissue-engineered bones (TEBs) for clinical applications lies in the constraints associated with the source and availability of autologous mesenchymal stem cells (MSCs) derived from the bone marrow, which creates a bottleneck. While allogeneic MSCs have shown promise in TEB applications, their ability to promote bone growth is notably diminished because of the inflammatory reaction at the transplant site and the inherent immune response triggered by allogeneic MSCs. Hence, there is a pressing need to develop methods that enhance the osteogenic differentiation of allogeneic MSCs during transplantation. Previous studies have found that IL-17 is a key proinflammatory factor in initiating inflammation and cascade amplification in the early stages of an inflammatory response, and proinflammatory cytokines such as TNF-*α* and IL-17 can inhibit the osteogenic differentiation of MSCs in an immune environment. In this study, MSCs expressing HVEM were successfully constructed by viral transfection and further reconfirmed that IL-17 can inhibit the in vivo and in vitro osteogenesis of allogeneic MSCs through in vitro experiments and mouse calvarial bone defect (diameter about 3 mm) model, while MSCs that express herpesvirus-entry mediator (HVEM) exhibit the capacity to suppress immune responses and sustain strong osteogenic potential. We further pointed out that the mechanism by which HVEM promotes the osteogenesis of allogeneic MSCs is related to its inhibition of the I*κ*B kinase (IKK)-NF-*κ*B signaling pathway activated by IL-17 in the immune environment, which can significantly inhibit the ubiquitination and degradation of *β*-catenin in MSCs induced by the IKK-NF-*κ*B pathway, upregulate the expression of *β*-catenin, and promote bone formation. Hence, this research provides an initial connection between the Wnt/*β*-catenin signaling pathway and the IKK-NF-*κ*B pathway during allogeneic MSC transplantation, offering new avenues for investigation and establishing a theoretical foundation for the potential use of HVEM-expressing MSCs in clinical treatments for bone defects.

## 1. Introduction

The field of bone defect repair has seen significant success in tissue-engineered bones (TEBs) using autologous bone marrow mesenchymal stem cells (auto-MSCs) as the primary cell source [1–3]. The clinical utilization of auto-TEB is somewhat restricted due to the limitations associated with auto-MSCs, particularly in terms of their quality and availability for preconstructing bone in sufficient quantities [4]. In contrast, allogeneic mesenchymal stem cells (allo-MSCs) have garnered increasing attention in recent years due to their favorable osteogenic capabilities [4–7]. However, it is worth noting that certain studies have indicated that allo-MSCs can trigger immune rejection and consequently result in graft failure [8, 9]. A growing body of evidence suggests that proinflammatory cytokines such as TNF-*α* and IL-17 have the capacity to impede the osteogenic differentiation of MSCs and the process of bone formation within an immune environment [10–13]. B and T lymphocyte attenuator (BTLA) and its ligand herpesvirus-entry mediator (HVEM) serve as crucial negative costimulatory signaling molecules within immune cells [14]. In previous studies, we successfully transfected the HVEM-Ig gene into MSCs and confirmed that MSCs expressing HVEM (MSCs-HVEM) were capable of suppressing immune responses and preserving the ability of MSCs to undergo osteogenic differentiation in vitro [15]. Furthermore, in mice, TEB-HVEM exhibited more favorable bone formation outcomes compared to TEB. Nonetheless, the precise mechanism through which MSCs expressing HVEM suppress the immune response and facilitate osteogenesis remains to be fully understood.

In our preliminary in vitro experiments, we observed that the presence of inflammatory cytokines, specifically IL-17, hindered the osteogenic differentiation of MSCs. We have indications that HVEM could potentially counteract this effect, and this reversal might be associated with its ability to either suppress IL-17 secretion or modulate immune activation pathways [15]. The transcription factor nuclear factor kappa B (NF-*κ*B) plays a major role in inflammation and host immune response. It can be activated by I*κ*B kinase (IKK) through a process involving the phosphorylation and subsequent degradation of I*κ*Bs [16, 17]. Studies have shown that IL-17 has the capacity to trigger the activation of I*κ*B kinase (IKK)-NF-*κ*B, which, in turn, can compromise the osteogenic differentiation of MSCs. On the contrary, the inhibition of IKK-NF-*κ*B can significantly enhance the osteogenesis of MSCs. Moreover, the activation of IKK-NF-*κ*B can induce ubiquitination and degradation of *β*-catenin by inducing Smurf1 and Smurf2 [13]. Other studies have shown that the number of IL-17-producing ROR*γ*t^+^CD27^−^*γδ*T cells, associated with autoimmune diseases, increased significantly in BTLA knockout mice [18]. The production of IL-17 was also significantly increased in response to IL-7 in BTLA-deficient *γδ*T cells [19]. As a result, we postulated that the beneficial effect of HVEM on promoting the osteogenic differentiation of MSCs by suppressing the inflammatory response might be intricately linked to the roles of IL-17 and NF-*κ*B pathways. In our current research, we have extended our investigations and provided additional confirmation that IL-17 indeed has the capacity to impede the osteogenic differentiation of MSCs. Furthermore, we have demonstrated that HVEM can counteract this inhibitory effect through both transgenic and chemical interventions. Additionally, our findings suggest that HVEM's mechanism of enhancing the osteogenic potential of MSCs may involve the inhibition of the NF-*κ*B pathway upregulation triggered by IL-17, as well as the reduction in the degradation of *β*-catenin. Our findings could offer valuable theoretical support for the potential clinical use of TEB-HVEM in the treatment of bone defects.

## 2. Materials and Methods

### 2.1. Isolation and Culture of Mouse Mesenchymal Stem Cells (mMSCs)

The animal experiments were approved by the Laboratory Animal Welfare and Ethics Committee of the Army Medical University (Ethics approval number: AMUWEC20226261). The mMSCs were isolated and cultured following established procedures, as previously described [15]. Briefly, the bone marrow was extracted from the femur and tibia of C57BL/6 mice. The marrow was flushed out using a syringe with C57BL/6 mouse MSC complete medium, which contained 1% penicillin-streptomycin, 1% glutamine, and 10% fetal bovine serum (FBS) sourced from Cyagen Biosciences, China (#MUXMX-90011). After centrifugation at 1000 rpm for 10 minutes and cell counting, the cells were plated onto 6-well plates, and the nonadherent cells were eliminated by changing the medium after 48 hours. The culture medium was refreshed every 3 days, and subculturing was performed when the cells reached 80–90% confluence. For flow cytometry analysis, the third passage cells were utilized, which were detached using 0.25% trypsin (#SH30042.01, HyClone, USA) (Sca-1^+^CD29^+^CD31—cell population was identified as MSCs). These cells were then utilized in subsequent experiments.

Professor Weifeng He (Burn Institute, State Key Laboratory, Southwest Hospital of Army Medical University, China) kindly provided us with recombinant adenoviruses that carried the HVEM gene along with the enhanced green fluorescent protein (EGFP) as a generous gift. MSCs expressing HVEM were generated, as previously expounded [15]. In short, MSCs were infected with recombinant Ad-EGFP-HVEM at a titer of 7 × 10^8^ U/ml for 24 hours. The transfection efficiency was assessed through the use of fluorescence microscopy and Western blot analysis. Through fluorescence microscope observation, the number of cells (i.e., positive cells) emitting strong green fluorescence signals under at least 3 different fields of view was counted, and the proportion of positive cells was calculated. Western blotting detected the expression of HVEM in MSCs 24 hours after transfection, using ordinary untransfected MSCs and Ad-EGFP adenovirus-transfected MSCs as controls, and repeated for three times.

### 2.2. Effect of IL-17 on the Osteogenic Differentiation of Both MSCs and MSCs Expressing HVEM

To delve deeper into the impact of IL-17 on the osteogenic differentiation of MSCs, we cultivated MSCs and HVEM-expressing MSCs (MSCs-HVEM) in 6-well plates (1 × 10^5^ cells/well). These cells were cultured in the osteogenic differentiation medium (OriCell C57BL/6 MSC Osteogenic Differentiation Medium, Cyagen Biosciences, China) in the presence or absence of 50 ng/mL recombinant mouse IL-17A (#7956-ML, R&D Systems, USA). The medium was replaced every 3 days. After 9 days, alkaline phosphatase (ALP) staining was carried out using a BCIP/NBT alkaline phosphatase assay kit (#3206, Beyotime Biotechnology, China). After 12 days, calcium nodules were stained using the Alizarin Red S staining method (#MUBMX-90021, Cyagen Biosciences, China), following the instructions provided by the manufacturer. ImageJ software was used to analyze the data. In short, after importing the images into the ImageJ software, the images were converted to 8-bit format, then the gray value was converted to OD value, the measurement parameters were set, the positive areas were selected by adjusting the threshold, and the specific value obtained by using the Analyze-Measure function was the integrated optical density value (IOD).

### 2.3. Western Blotting Analysis

The expression of osteogenic markers (Collagen I, Runx2), as well as signaling pathway-related proteins (I*κ*B*α*, p-I*κ*B*α*, p65, p-p65, and *β*-catenin), in MSCs after IL-17 treatment was detected by Western blotting. After treatment at the specified time points, the entire cell population within each well was lysed using radioimmunoprecipitation assay (RIPA) lysis buffer (Sigma, USA) containing 1% phosphatase inhibitor, 0.1% protease inhibitor, and 0.5% 100 mM phenylmethylsulfonyl fluoride (Roche, Swiss). The supernatant was harvested, and the protein concentration was determined through a BCA assay. Subsequently, equal quantities of protein (20 *μ*g) were separated by 10% sodium dodecyl sulfate (SDS)-polyacrylamide gel electrophoresis (PAGE) and then transferred onto 0.2 *μ*m polyvinylidene fluoride (PVDF) membranes (Millipore, USA). The membranes were blocked at room temperature for 2 hours and then subjected to overnight incubation with primary antibodies in a solution containing 3% bovine serum albumin (BSA) at 4°C. The primary antibodies included anti-COLI (#72026, Rabbit mAb, 1 : 1000, CST, USA), Runx2 (#12556, Rabbit mAb, 1 : 1000, CST, USA), I*κ*B*α* (#9242, Rabbit mAb, 1 : 1000, CST, USA), p-I*κ*B*α* (#2859, Rabbit mAb, 1 : 1000, CST, USA), p65 (#8242, Rabbit mAb, 1 : 1000, CST, USA), p-p65 (#3033, Rabbit mAb, 1 : 1000, CST, USA), *β*-catenin (#ab32572, Rabbit mAb, 1 : 5000, Abcam, UK), and *β*-tubulin (#KM9003T, Mouse mAb, 1 : 5000, Sungene Biotech, China) and GAPDH (#KM9002T, Mouse mAb, 1 : 5000, Sungene Biotech, China). Subsequently, the membranes underwent five washes with TBST (Tris-buffered saline containing 0.1% Tween-20) and were then incubated with corresponding fluorescent secondary antibodies (Sungene Biotech, China) for 1 hour at room temperature. Images were obtained and analyzed using a chemiluminescence kit (Amersham Biosciences, UK) and Quantity One software.

### 2.4. Calvarial Bone Defect Model and Histological Evaluation

**Figure 1:**
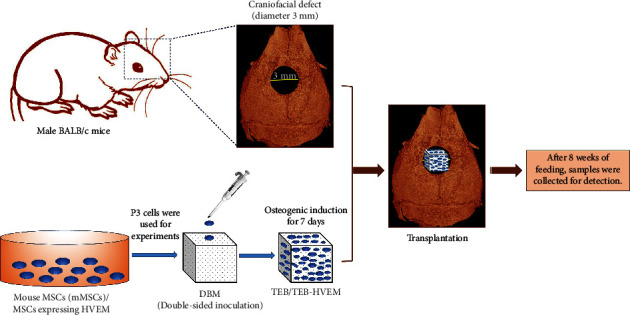
In vivo experimental design in mice: established a BALB/c mouse calvarial bone defect model (with a diameter of about 3 mm, without damaging the dura and brain tissue), and constructed tissue-engineered bone (TEB) based on different seed cells (MSCs or MSCs-HVEM), and different TEBs were implanted into the calvarial bone defect area and fed for 8 weeks for subsequent testing.

The TEBs were constructed, as previously described [15]. In simple terms, 2 × 10^4^ mouse MSCs were inoculated on both upper and lower sides of decalcified bone matrix (DBM) to construct TEBs and then cultured with osteogenic induction medium for 7 days. To prepare HVEM-expressing TEBs, MSCs in above TEBs were transfected with Ad-EGFP-HVEM for 24 hours on the 6th day of osteogenic induction culture. In this experiment, twelve adult male BALB/c mice, sourced from the Laboratory Animal Center of the Army Medical University, approximately 8 weeks old, were randomly divided into four groups using numbering and drawing lots. These groups received the following transplantations: (1) DBM/MSCs, (2) DBM/MSCs + IL-17, (3) DBM/MSCs-HVEM, and (4) DBM/MSCs-HVEM + IL-17, with each group consisting of three mice. In short, BALB/c mice were anesthetized with 0.15 mL 0.1% pentobarbital sodium through intraperitoneal injection. After the anesthesia was effective, the mice were placed in a prone position and fixed on a small animal operating table. The head surgery area was routinely prepared for skin preparation, disinfected with iodine, and covered with sterile sheets. A longitudinal scalp incision was made at the center of the frontal calvarial bone, extending backward from the nasal bone. Following this, full-thickness flaps were raised. Following the complete shaving of the periosteum covering the surface of the calvarial bone, a bone defect with an approximate diameter of 3 mm was meticulously created at the midline of the skull, utilizing a grinding drill to prevent any damage to the dura or brain. The four aforementioned materials were then implanted into the created defect sites (Figure 1). These transplantations were performed both with and without IL-17 (100 ng) in separate groups. At the 8-week postoperation mark, all the mice were euthanized by intermittent slow intraperitoneal injection of high-concentration pentobarbital sodium solution (3%) and their calvarial bones were excised. The mice corpses were sealed and packaged in specialized biosafety bags, and stored in a −20°C freezer for centralized freezing. Later, they were uniformly incinerated for harmless treatment. These calvarial bone specimens were subsequently fixed in 4% paraformaldehyde for a period of one week. Following fixation, microcomputed tomography (micro-CT, BRUKER, Belgium) scanning was carried out to assess the overall morphology of the new bone within the defect sites and to analyze parameters, such as bone mineral density (BMD), bone volume to total bone volume ratio (BV/TV), trabecular number (Tb.N), and trabecular separation (Tb.Sp). Subsequently, the calvarial bones were subjected to decalcification in a 10% EDTA solution for a period of two weeks at 4°C. Following decalcification, they were embedded in paraffin. The samples were sectioned into 5 *μ*m-thick slices along the sagittal plane for Masson trichrome staining. To further investigate the mechanism, immunohistochemistry was conducted to examine the expression of *β*-catenin in the transplantation area. This process involved the use of SABC IHC kits from Zhongshan Corporation, China. The sections were incubated with primary antibodies against *β*-catenin (#ab32572, Rabbit mAb, 1 : 250, Abcam, UK) at 4°C overnight, following the manufacturer's instructions. Nuclei were then counterstained with hematoxylin, and images were captured using a Leica Microsystems microscope (DFC300 FX, Switzerland).

### 2.5. Statistical Analysis

The data presented in this study were derived from three distinct independent experiments and were plotted as mean ± standard deviation (SD). If the variances were uneven, the data were processed using logarithmic transformations or nonparametric tests. If the variance met homogeneity, then one-way analysis of variance (ANOVA) was performed using the GraphPad Prism 6.0 statistical software package (GraphPad, USA). In all tests, *p* < 0.05 was considered statistically significant. If the results were statistically significant, the Fisher LSD test was used to compare the differences between any two groups. Student's *t*-test was used to compare the two groups of data, and the *p* < 0.05 was considered to be statistically significant. All statistical analyses were performed using SPSS software (12.0, USA). The data points on each plot represented a single sample, the center line represented the mean, and the error bar represented the SD.

## 3. Results

### 3.1. Successful Construction of MSCs Expressing HVEM and TEB

**Figure 2:**
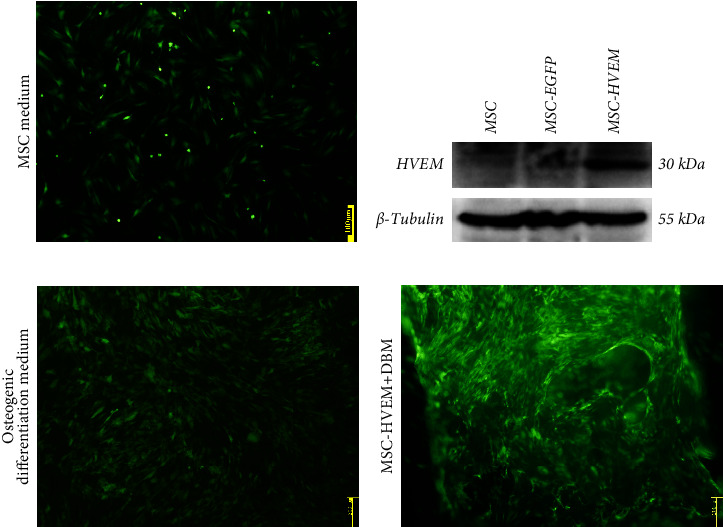
Successfully constructed MSCs expressing HVEM (MSCs-HVEM) using Ad-EGFP-HVEM recombinant adenovirus. (a) Under a fluorescence microscope, it can be observed that MSCs-HVEM cells had good morphology, adherent growth, and emitted strong green fluorescence. (b) The WB detection results confirmed that the transfected MSCs expressed high levels of HVEM protein, while conventional MSCs and recombinant Ad-EGFP adenovirus-transfected MSCs did not express HVEM protein. (c) MSCs-HVEM exhibited a bundle-like or vortex-like growth pattern consistent with conventional MSCs after osteogenic induction culture, and their state was good (×40). (d) The TEB constructed based on MSCs-HVEM emitted strong green fluorescence, and the adhesion of MSCs-HVEM to scaffold material was determined by the precipitation method, and the results showed that the cells adhered well to the scaffold material. EGFP, enhanced green fluorescent protein. HVEM, herpesvirus-entry mediator.

MSCs that had been cultured up to the third generation were transfected with recombinant Ad-EGFP-HVEM at a titer of 7 × 10^8^ U/ml for a duration of 24 hours. Following the transfection, the culture medium was replaced to establish MSCs that express HVEM. Upon examination under fluorescence microscopy, it was evident that the MSCs maintained good cell morphology characterized by a spindle shape, a prominent nucleus-to-cytoplasm ratio, robust adherence, and the ability to undergo continuous subculture (Figure 2(a)). The results of Western blot (WB) analysis demonstrated that the transfected MSCs exhibited elevated levels of HVEM protein expression, whereas both conventional MSCs and MSCs transfected with recombinant Ad-EGFP did not exhibit HVEM expression (Figure 2(b)), indicating the successful construction of MSCs expressing HVEM. Furthermore, MSCs expressing HVEM exhibited a growth pattern characterized by bundle-like or vortex-like structures, which was consistent with the typical growth pattern of conventional MSCs during osteogenic induction culture. Their overall condition remained favorable (Figure 2(c)). Moreover, an abundance of green fluorescent-labeled MSCs was observed on the TEB constructed using MSCs expressing HVEM. These cells exhibited strong adherence to the scaffold material, making them suitable for subsequent in vivo experiments related to bone defects (Figure 2(d)).

### 3.2. IL-17 Inhibited the Osteogenic Differentiation of MSCs

**Figure 3:**
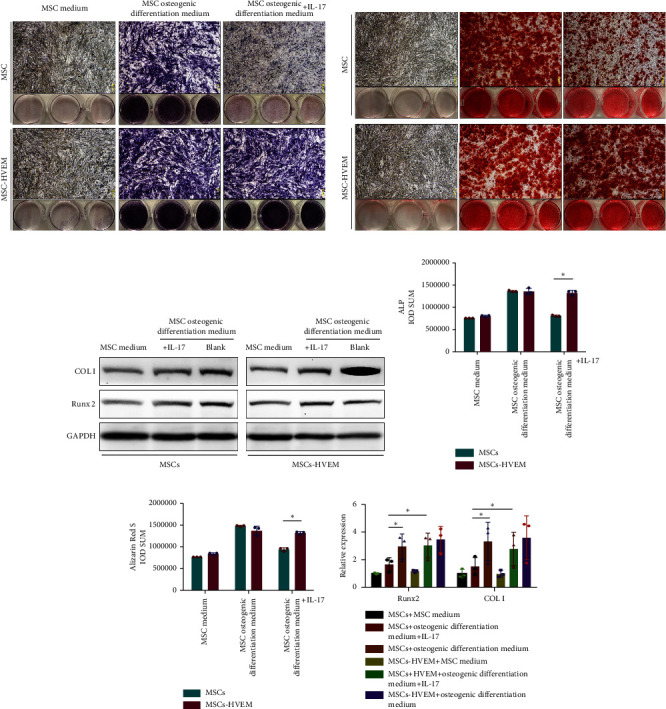
IL-17 inhibited the osteogenic differentiation of MSCs. MSCs expressing HVEM not only maintained their osteogenic differentiation ability, but also significantly enhanced the inhibitory effect of IL-17 on osteogenic differentiation of MSCs. (a) On the 9th day, ALP staining was performed on the samples fixed with paraformaldehyde in each group. (b) On the 12th day, samples from each group were stained with Alizarin Red S staining (calcium nodules). (d) and (e) The IOD (integral optical density) sum of ALP and Alizarin red S staining was quantified using ImageJ (*n* = 3). (c) and (f) Expression of osteogenic markers detected by Western blotting. Relative protein expression of COLI and Runx2 (relative to expression in MSCs in MSC conventional culture medium without osteogenic induction and IL-17). ALP, alkaline phosphatase; COLI, collagen type I; Runx2, Runt-related transcription factor 2. Data are reported as mean ± SD. ^∗^*p* < 0.05 (Student's *t*-test).

In order to assess whether HVEM can counteract the inhibitory impact of IL-17 on the osteogenic differentiation of MSCs, we subjected mMSCs and MSCs expressing HVEM to IL-17 treatment at a concentration of 50 ng/mL for a duration of 9 and 12 days, respectively. As shown in Figures 3(a), 3(b), 3(d), and 3(e), both ALP staining and Alizarin Red S staining revealed that IL-17 had an inhibitory effect on the osteogenic differentiation of MSCs. Importantly, the presence of HVEM in MSCs not only preserved their osteogenic differentiation ability but also notably mitigated the inhibitory impact of IL-17 on MSC osteogenesis (MSCs + IL-17 vs MSCs-HVEM + IL-17: ALP *p*=0.00006, Alizarin Red S *p*=0.0002). Similar trends were also observed in the expression of osteogenic markers (Figures 3(c) and 3(f)). Western blot results indicated that the presence of IL-17 led to a reduction in the expression of Runx2 and COL I in MSCs. In contrast, the expression of these markers in MSCs expressing HVEM remained largely unaffected (MSCs + IL-17 vs MSCs-HVEM + IL-17: Runx2 *p*=0.029, COL I *p*=0.043).

### 3.3. HVEM Inhibited the Upregulation of the NF-*κ*B Pathway Induced by IL-17

**Figure 4:**
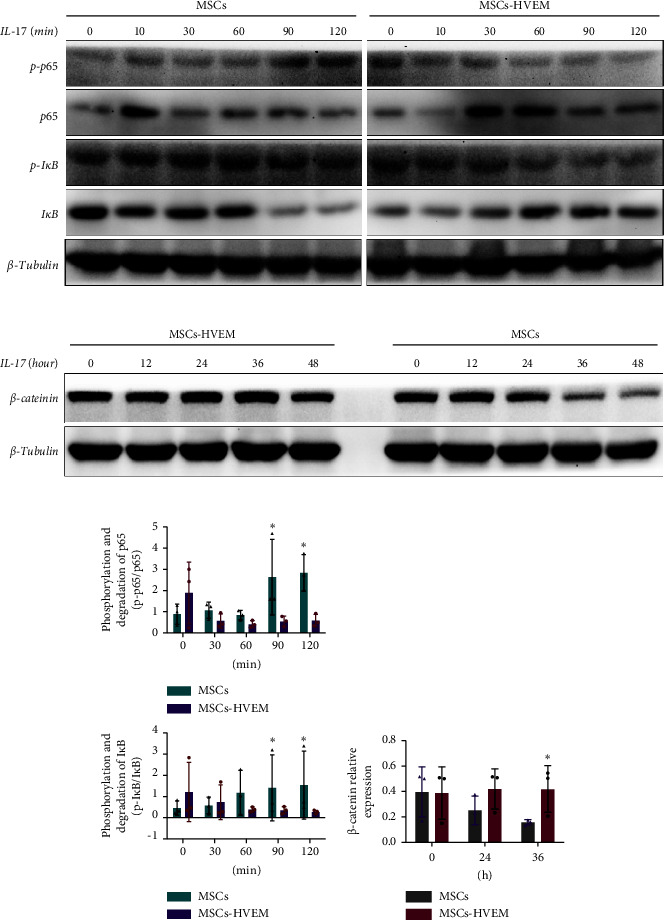
HVEM inhibited the upregulation of NF-*κ*B signaling pathway and downregulation of *β*-catenin expression induced by IL-17. (a) The expression of NF-*κ*B pathway-related proteins (such as I*κ*B*α*, p-I*κ*B*α*, p65, and p-p65) in MSCs and MSCs-HVEM after IL-17 treatment was detected by Western blotting. (b) Western blotting was used to detect the expression of *β*-catenin in MSCs and MSCs-HVEM treated with IL-17. (c) Histogram showing the degree of phosphorylation and degradation of I*κ*B and p65. (d) Relative protein expression of *β*-catenin. Data are reported as mean ± SD. ^∗^*p* < 0.05 (Student's *t*-test).

Considering that IL-17 has the potential to activate IKK-NF-*κ*B and damage the osteogenic differentiation of MSCs, both MSCs and MSCs expressing HVEM were subjected to IL-17 treatment at a concentration of 50 ng/mL for varying durations, specifically 0, 10, 30, 60, 90, and 120 minutes. The results showed that IL-17 rapidly induced the degradation and phosphorylation of I*κ*B*α* in MSCs, which is a direct target gene of NF-*κ*B. Furthermore, it was observed that IL-17 rapidly induced phosphorylation of p65 at the S536 site, as evidenced by the use of anti-phospho-p65-S536 antibodies. Conversely, MSCs expressing HVEM exhibited a notable capacity to counteract the degradation of I*κ*B*α* induced by IL-17 (Figures 4(a) and 4(c)). This implies that as the duration of IL-17 treatment increased, the degradation of I*κ*B*α* progressively diminished under the influence of HVEM. Similarly, HVEM showed a similar alleviating effect on IL-17-induced p65 degradation (MSCs vs MSCs-HVEM (120 mins): p-p65/p65 *p*=0.0273, p-I*κ*B/I*κ*B *p*=0.0288). On the other hand, in order to further understand how IL-17 activates the NF-*κ*B pathway and inhibits the osteogenic differentiation of MSCs, we induced osteogenesis in both MSCs and MSCs expressing HVEM in the presence of IL-17 (50 ng/mL) for varying durations, specifically 0, 12, 24, 36, and 48 hours. The WB results showed that as the duration of IL-17 treatment extended, the expression of *β*-catenin in MSCs progressively decreased. In contrast, the expression of *β*-catenin in MSCs expressing HVEM exhibited no significant alteration (Figures 4(b) and 4(d). MSCs vs MSCs-HVEM (36 hours): *p*=0.0359). These findings suggest that HVEM plays a critical role in preserving the expression of *β*-catenin and can markedly enhance the osteogenic differentiation of MSCs, particularly in an immune-activated environment.

### 3.4. HVEM Reversed the Inhibitory Effect of IL-17 on the Osteogenic Differentiation of MSCs and Promoted the Repair of Mouse Calvarial Bone Defect

**Figure 5:**
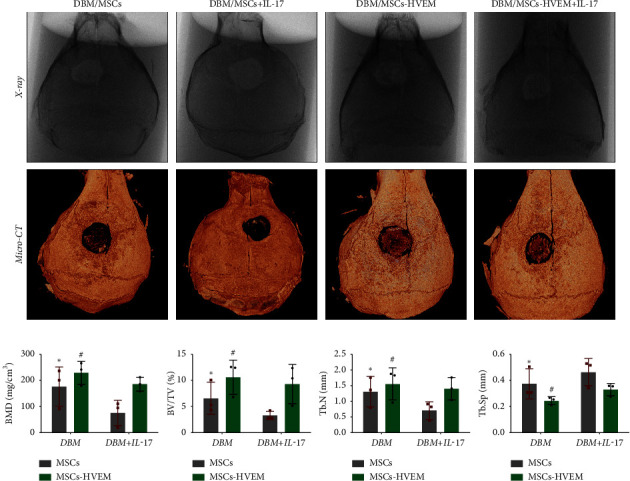
Morphological analysis of new bone formation in calvarial bone defects. (a) X-ray and micro-CT images of the calvarial bone defects in four groups at 8 weeks after implantation. (b) Microarchitecture parameter analyses of the new bone formation areas. BV/TV, bone volume fraction (bone volume to total bone volume ratio); Tb.N, trabecular number; BMD, bone mineral density; Tb.Sp, trabecular separation; CT, computed tomography; DBM, demineralized bone matrix. Data are reported as mean ± SD. ^∗^*p* < 0.05, ^#^*p* ≥ 0.05 (Student's *t*-test).

**Figure 6:**
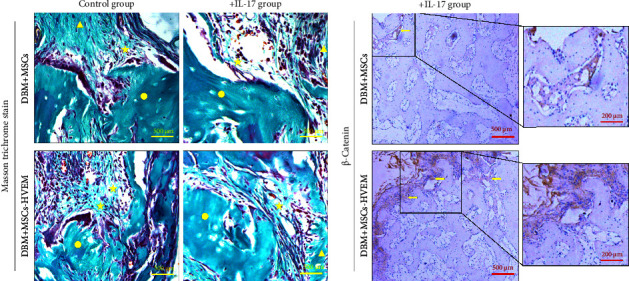
(a) Histological analysis of newly formed bone tissue in mouse calvarial bone defect area using Masson's trichrome staining. Yellow circle: DBM. Yellow asterisk: new bone callus formation area. Yellow triangle: mice calvarial bone tissue. (b) Immunohistochemical staining was used to detect the expression of *β*-catenin in the bone defect area. The yellow arrow showed the *β*-catenin expression region.

A mouse skull defect, approximately 3.0 mm in diameter, was effectively created, and TEBs derived from allogeneic MSCs and MSCs expressing HVEM were transplanted into the bone defect region. After an 8-week period of observation, morphological evaluations were conducted. X-ray imaging and micro-CT three-dimensional reconstructions revealed the presence of new callus formation in both the normal TEB group (DBM/MSCs) and the TEB group expressing HVEM (DBM/MSCs-HVEM) (Figure 5(a)). Notably, the area of new bone formation in the DBM/MSCs-HVEM group was significantly larger compared to that in the DBM/MSCs group, indicating superior osteogenic capability in the former. Simultaneously, it was evident that the addition of IL-17 to the transplantation site inhibited the bone-forming ability of the regular TEB and resulted in a notable reduction in the area of new callus formation. In contrast, the TEB group expressing HVEM did not exhibit a significant decrease in new bone formation, maintaining its superior performance compared to the regular TEB group (Figure 5(a)). A more detailed analysis of bone micromorphological parameters in each group was conducted using CTvox software. This analysis encompassed parameters, such as BMD, BV/TV, Tb.N, and Tb.Sp. The DBM/MSC-HVEM group displayed better values in the bone callus compared to the DBM/MSC group (Figure 5(b)). Even with the introduction of IL-17 into the transplantation site, the bone callus parameters in the DBM/MSC-HVEM group continued to exhibit significant superiority over those in the DBM/MSC group (Figure 5(b), DBM/MSCs + IL17 vs DBM/MSC-HVEM + IL17: BMD *p* *=* 0.0133, BV/TV *p*=0.0264, Tb.N *p*=0.0282, Tb.Sp *p*=0.0459). Additional examination of the extent of new bone formation in the graft area, utilizing Masson's trichrome staining, corroborates the same pattern observed in X-ray and micro-CT images, as described earlier (Figure 6(a)). This comprehensive evidence underscores that in the context of allogeneic MSC tissue engineering for bone grafting, MSCs expressing HVEM exhibited superior osteogenic potential and could markedly ameliorate the inhibitory impact of IL-17 on MSC osteogenesis. Moreover, immunohistochemical staining revealed a substantial reduction in the expression of *β*-catenin in the transplantation area of the DBM/MSCs group when exposed to IL-17 (Figure 6(b)). In contrast, the DBM/MSC-HVEM group exhibited a noteworthy upregulation of *β*-catenin expression in the same transplantation area (Figure 6(b)). This observation highlights the significant role of HVEM in mitigating the downregulation of *β*-catenin expression induced by IL-17 and promoting the process of bone repair.

## 4. Discussion

TEB transplantation based on allogeneic MSCs holds great potential as a therapeutic approach for bone regeneration and repair. The osteogenic differentiation of MSCs is intricately controlled by the interplay of mechanical and molecular cues from the surrounding extracellular environment [20–24]. In the realm of bone regeneration studies, bone defects or damaged tissues frequently experience inflammatory responses, which are often accompanied by atypical expression of inflammatory mediators [25–27]. Furthermore, the intrinsic immunogenicity associated with allogeneic MSCs frequently provokes immune rejection reactions when these cells are transplanted into the body. This immune response can result in an elevated failure rate for allogeneic MSC transplantation. Numerous studies have demonstrated that within the immune environment, inflammatory cytokines and proinflammatory factors, such as IL-17 and TNF, have the capacity to hinder the osteogenic differentiation and osteogenic potential of MSCs [10–13]. Nevertheless, the precise mechanism through which these factors inhibit MSC differentiation remains incompletely understood. Furthermore, despite substantial advancements in our understanding of how MSCs regulate T cell and macrophage functions, there is limited research regarding how local inflammation impacts MSC-mediated bone regeneration and repair in vivo. Elevated expression levels of TNF and IL-17 have been detected in various chronic inflammatory bone diseases, including arthritis, osteoporosis, periapical periodontitis, and periodontal disease [12, 26, 28–30]. Hence, to enhance the osteogenic potential of allogeneic MSCs within an immune-activated microenvironment, it is of paramount importance to devise novel strategies that can surmount immune rejection and attain successful regenerative therapy. In this experiment, as part of the in vitro osteogenic differentiation of MSCs, we treated MSCs and MSCs expressing HVEM with mouse recombinant IL-17. The findings once more reaffirmed the earlier conclusion that IL-17 had an inhibitory impact on the osteogenic differentiation of MSCs, whereas MSCs expressing HVEM were able to counteract this inflammatory effect.

The transcription factor nuclear factor *κ*B (NF-*κ*B) serves as a central regulator in the body's inflammatory response and host immune response. NF-*κ*B can be activated by proinflammatory cytokines like TNF and IL-17, as well as by factors, such as LPS and viral DNA, particularly in cases of inflammatory diseases and tissue damage. The I*κ*B kinase (IKK) complex plays an important role in the activation of NF-*κ*B by phosphorylating and degrading I*κ*Bs [16, 17, 31–34]. Recent investigations have revealed that the IKK-NF-*κ*B signal transduction pathway exerts an inhibitory influence on the osteogenic process in differentiated osteoblasts [13]. Moreover, it has been observed that a time-specific and stage-specific inhibition of the IKK-NF-*κ*B signaling pathway in differentiated osteoblasts can substantially enhance the formation of bone matrix and mineral density during postnatal growth [10, 12].

In this experiment, we also confirmed that the proinflammatory cytokine IL-17 can activate the IKK-NF-*κ*B signaling pathway and hamper the osteogenic differentiation of MSCs, while HVEM can significantly downregulate this pathway, ultimately promoting osteogenesis. Considering the inhibitory effect of HVEM on NF-*κ*B in inflammation and infection, we speculate that targeting the IKK-NF-*κ*B pathway could open up novel avenues for enhancing bone regeneration and repair strategies. This also suggests a promising direction for investigating the mechanism by which HVEM suppresses immune responses while fostering bone formation. However, these findings appear to be in contrast with earlier research, as two prior studies suggested that TNF activates NF-*κ*B and promotes the osteogenic differentiation of bone marrow mesenchymal stem cells [35, 36]. At present, we are unable to provide a definitive explanation for this discrepancy. Nevertheless, it is important to note that these two earlier studies did not conduct in vivo experiments to validate their findings. Additionally, it is worth mentioning that Kaneki et al. discovered that TNF inhibits bone formation in the body [37]. Chen et al. similarly demonstrated that DNA damage can impede the osteogenic differentiation of MSCs. This process can concurrently activate NF-*κ*B both in vitro and in vivo, which ultimately accelerates the aging of bone tissues [38].

The primary constraint of this study is that we have solely investigated and validated, in isolation, the ability of HVEM to inhibit the activation of the NF-*κ*B signaling pathway induced by IL-17 and effectively curb the degradation of *β*-catenin within the Wnt signaling pathway. Nonetheless, the interplay between the Wnt and NF-*κ*B signaling pathways in MSCs remains uncharted territory and has yet to be elucidated. Chang et al., employing gene chip techniques, discovered that the IKK-NF-*κ*B signaling pathway plays a role in promoting the degradation and ubiquitination of *β*-catenin by inducing Smurf2. Moreover, inhibiting the IKK-NF-*κ*B pathway can counteract the decrease in Smurf2 expression in MSCs induced by IL-17. This effect may be attributed to NF-*κ*B's direct binding to the promoter region of Smurf2, which in turn regulates Smurf2 expression in MSCs [13]. This discovery provides an initial connection between the Wnt and NF-*κ*B signaling pathways, which are associated with HVEM's role in enhancing the osteogenic differentiation of MSCs. In conclusion, our results provide a theoretical basis for the use of TEB-HVEM in the treatment of clinical bone defects and provide a new way to solve the possible immune rejection after bone repair material transplantation. Further exploration and understanding of the relationship between Wnt and NF-*κ*B signaling pathway is crucial to continuously optimize the osteogenic effect of TEB-HVEM, which is also one of our future research priorities.

## Data Availability

The image data used to support the findings of this study are included within the article.
